# Retrospective analysis of infectious agents in swine abortion materials in the years 2021 to 2023

**DOI:** 10.1007/s11259-026-11252-x

**Published:** 2026-05-29

**Authors:** Henning Bischoff, Marius Beumer, Carina Helmer, Ines Spiekermeier, Juhle-Marijke Buch, Christiane Hundehege, Theresa Menke, Esther Humann-Ziehank, Isabel Hennig-Pauka

**Affiliations:** 1 SAN Group Biotech Germany, Mühlenstraße 13, Höltinghausen, 49685 Germany; 2 Present Address: Genovo GmbH, Kirchstr. 3, Grossenkneten, 26197 Germany; 3 LABVETCON, Föhrenkamp 20, Burgdorf, 31303 Germany; 4 Field Station for Epidemiology, University of Veterinary Medicine Hannover Foundation, Büscheler Straße 9, Bakum, 49456 Germany

**Keywords:** Swine, Abortion, Prevalence, PRRSV, Betaarterivirus suid, Diagnostic, Real-time PCR

## Abstract

Abortions in swine are caused by infectious and non-infectious factors and play an important economic role in the swine industry. Molecular biological testing for relevant pathogens is routine in state-of-the-art diagnostics. The aim of this study was to assess the prevalence of pathogens associated with abortions in central Europe to prioritize differential diagnoses. We report the detection rates of such pathogens in aborted material (fetuses, placenta) from 199 abortion cases sent to AniCon Labor GmbH/SAN Group Biotech Germany GmbH from January 2021 to July 2023. A key panel of pathogens that were tested in most abortion cases emerged, consisting of porcine reproductive and respiratory syndrome virus (PRRSV), Chlamydiaceae spp., porcine circovirus 2 (PCV2), porcine parvovirus (PPV) and Leptospira spp.. At least one of these pathogens was detected by real-time PCR in 38 of the 199 cases (19.1%). PRRSV (n=16) and Chlamydiaceae spp. (n=12) were most frequently found, whereas PCV2 (n=6), PPV (n=4) and Leptospira (n=2 cases) were detected less frequently. In 14 cases, potentially relevant pathogens outside the key panel were detected by molecular biological or culture methods. Three of those cases were additionally positive for a pathogen in the key panel. In total, pathogens associated with abortions were detected in 24.6% of cases. It is hypothesized that a more comprehensive diagnostic approach will lead to more frequent detection of pathogens not regularly tested by PCR in routine diagnostics.

**Supplementary Information:**

The online version contains supplementary material available at 10.1007/s11259-026-11252-x.

## Introduction

Abortion diagnostics in swine are challenging for practitioners due to a wide range of potential infectious and non-infectious etiological factors. Pathogens can either infect placenta and fetus or cause systemic disease of the sow, in which case concomitant increased cytokine levels can induce a regression of the corpora lutea via a variety of pathways, resulting in a decrease in progesterone levels that can lead to abortion (Maes et al. 2023).

Studies of infectious agents in aborted material sent for routine diagnostics resulted in varying prevalences of 27–42% (Nathues et al. 2011; Donneschi et al. 2024). Usually, cases with no pathogen detection are attributed to non-infectious causes.

A key panel of five pathogens is regularly tested in abortion material in diagnostic laboratories in Germany: porcine reproductive and respiratory syndrome virus (PRRSV), Chlamydiaceae spp., porcine circovirus 2 (PCV-2), porcine parvovirus (PPV) and *Leptospira.* In other regions of the world, additional pathogens such as *suid herpesvirus 1*, transmissible gastroenteritis virus, classical swine fever virus, African swine fever virus, *Brucella suis* and many others, play important roles (Pozzi and Alborali 2012; Salogni et al. 2016).

The aim of this study was to analyze the prevalence of pathogens in swine abortion material (fetuses, placenta) in central Europe through retrospective analysis of diagnostic data obtained from routine diagnostic services at AniCon Labor GmbH (now SAN Group Biotech Germany GmbH) from January 2021 to July 2023.

## Materials and methods

### Samples and sampling

From January 2021 to July 2023, material from 199 cases of abortion was sent to the service laboratory AniCon Labor GmbH/SAN Group Biotech Germany GmbH. The sample material originated from a total of 143 production sites and consisted of aborted material (aborted fetuses and placenta) and occasionally (*n* = 11) accompanying blood, swab or oral fluid samples taken from the aborting sow. Most of these cases (*n* = 176) originated from Germany, others were sent from Ireland (*n* = 17), Austria (*n* = 3), Poland (*n* = 2), or Lithuania (*n* = 1). The organs of aborted fetuses and placentas were pooled from up to five individuals according to the diagnostic requests of the customer and depending on the tissue tropism of the different pathogens (Supplementary Table 1). One case was defined as one sample shipment from one herd. It is possible that one shipment contained sample material from more than one sow, and these incidents were summarized as one case. In total, 1629 samples were tested across the 199 cases originating from 143 farms.

### Nucleic acid extraction and real-time (RT-)PCR

Pooled organs were covered with 3–5 volumes of 154 mM NaCl and homogenized using stainless steel beads and a FastPrep-24™ tissue homogenizer (MP Biomedicals, Irvine, CA, USA). The homogenates were diluted 1:5 (v: v) in 154 mM NaCl, and nucleic acids were purified using the Kylt^®^ RNA/DNA Purification Kit HTP (SAN Group Biotech Germany GmbH, Höltinghausen, Germany) according to the manufacturer’s instructions. Accompanying blood samples were diluted 1:5 (v: v) with 154 mM NaCl and subsequently processed using the Kylt^®^ RNA/DNA Purification Kit HTP (SAN Group Biotech Germany GmbH, Höltinghausen, Germany). Swab samples were washed out by vortexing in 1 ml of 154 mM NaCl. Nucleic acids were extracted from the washout using the Kylt^®^ RNA/DNA Purification Kit HTP (SAN Group Biotech Germany GmbH, Höltinghausen, Germany).

The extracted nucleic acids were used in different real-time PCR kits listed in supplementary Table 2 according to the manufacturer’s instructions. PCRs were run on a CFX384 Touch real-time PCR detection system or a CFX96 Touch real-time PCR detection system (Bio-Rad Laboratories Inc., Hercules, CA, USA).

### Sanger sequencing and phylogenetic analysis of porcine reproductive and respiratory syndrome virus

Upon customer request, samples that tested positive for PRRSV were subjected to Sanger sequencing analysis. Complementary DNA (cDNA) was synthesized using qScript cDNA SuperMix (Quantabio LLC, Beverly, MA, USA). A partial sequence of open reading frame 5 was subsequently amplified using in-house primers and HotStarTaq Master Mix (QIAGEN GmbH, Hilden, Germany). Following PCR, DNA cleanup was performed using MagSi-NGSprepPlus Beads (magtivio BV, Nuth, Netherlands), and the DNA concentrations were measured spectrophotometrically. Sequencing reactions were prepared for sequencing at Eurofins Genomics GmbH according to the supplier’s guideline.

Resulting sequencing traces were evaluated using the SeqMan software in the Lasergene Molecular Biology Suite (DNAStar Inc., Madison, WI, USA). Obtained contigs were compared against internal sequence databases and the NCBI nucleotide database using BLAST (Altschul et al. 1990). Alignments and phylogenetic trees were constructed using the MegAlign Pro software in the Lasergene Molecular Biology Suite (DNAStar Inc., Madison, WI, USA).

### Bacterial culture

Swabs from the stomach contents and the livers of aborted fetuses were streaked onto chocolate blood agar with Vitox Supplement and various Columbia blood agar plates, either with *Staphylococcus epidermidis* as nurse bacterium, with neomycin, with gentamycin, and without supplement. Following a 48-hour incubation period at 37 °C, species identification was performed by MALDI-TOF on a Bruker microflex LT machine (Bruker Corporation, Billerica, MA, USA).

### Statistical approach

The analysis was descriptive in nature. For each pathogen, detection rates were calculated as the proportion of positive cases (see case definition) among all examined cases. Results are presented as absolute numbers and percentages. No inferential statistical tests were performed, as the study objective was limited to reporting detection frequencies.

## Results

### Detection rate of the key panel of pathogens

During the study period, 199 cases of swine abortions from 143 production sites were submitted for diagnostics, resulting in a total of 1629 organ pools tested according to customer orders. A key panel was tested in most cases, comprising PRRSV, PPV, PCV2, *Leptospira* and *Chlamydiaceae* spp. Samples from 38 cases tested positive for at least one pathogen from the key panel, resulting in a detection rate of 19,1%. Positive test results per case and per pathogen are shown in Table 1. Among these pathogens, PRRSV (8.8%) and Chlamydiaceae spp. (9.2%) were the most frequently detected. Two samples were positive for *Chlamydiaceae* spp. as well as for PRRSV.

In three additional cases, pathogens from the key panel were detected not in the aborted material but in additional samples from the aborting sow. Specifically, one blood sample and one oral sample tested positive for PRRSV, and one nasal swab tested positive for PPV. Due to the unclear significance of these findings in the mentioned matrices, these findings were excluded from the number of cases with detected pathogens.

**Table 1:** Detection rates of selected pathogens in abortion material (organ pools acc. to suppl. Table 1) and additional detections in accompanying samples from the aborting sow (displayed in brackets with sample matrix)

Pathogen	PRRSV	PPV	PCV2	Leptospira spp.	Chlamydiaceae spp.
Number of tested cases	181	180	176	150	131
No. of positive cases	16 (+ 2 oral fluid, blood)	4 (+ 1 nasal swab)	6	2	12
Rate of positives	8.8%	2.2%	3.4%	1.3%	9.2%

### Further diagnostic findings

Fifty-nine cases were examined for additional pathogens by PCR, specifically porcine circovirus 3 (PCV-3, *n* = 20), *Erysipelotrix rhusiopathiae* (*n* = 18), African swine fever virus (*n* = 13), *Mycoplasma suis* (*n* = 5) and Influenza virus A (IVA) (*n* = 3). Among these tests, two cases tested positive for PCV-3 (10%), and one case tested positive for *Mycoplasma suis*. In eleven out of 14 cases with bacteriological examination, bacteria potentially associated with reproductive disease were detected (Table 2). In total, potentially relevant pathogens not included in the key panel were detected in 14 cases. However, in three cases one of the key panel pathogens was found as well: In one sample PCV-2 was detected by real-time PCR and *Trueperella abortisuis* was isolated by cultural methods. Two further samples tested positive for PRRSV by Real-Time PCR and *Streptococcus suis* or *Escherichia coli* was isolated in cultural methods, respectively.

Overall, no pathogen was detected in the aborted material in 150 of the 199 cases (75.4%).

In nine cases, accompanying sample material from the sow was tested for IVA, with three of those samples yielding positive results.

**Table 2:** Detected bacterial species in the liver tissue or stomach of aborted fetuses from 14 cases

Case	Detected bacteria with correlation to reproductive symptoms	Further isolated bacteria
1	*Escherichia coli*,* Staphylococcus aureus*	*-*
2	*Escherichia coli*,* Streptococcus suis*	*Pasteurella multocida*
3	*-*	*Streptococcus dysgalactiae* subspecies *dysgalactiae*
4	*Escherichia coli*	*Glaesserella parasuis*
5	*Escherichia coli*	*Streptococcus dysgalactiae* subspecies *equisimilis*
6	*Escherichia coli*	*Lysinibacillus sphaericus*
7	-	*Streptococcus dysgalactiae subspecies dysgalactiae*
8	*Trueperella abortisuis*	-
9	*Streptococcus suis*	-
10	-	*Streptococcus dysgalactiae subspecies equisimilis*
11	*Escherichia coli*,* Staphylococcus hyicus*	*Enterococcus hirae*
12	*Escherichia coli*	*Clostridium perfringens*,* Enterococcus hirae*
13	*Escherichia coli*	-
14	*Escherichia coli*	*Streptococcus dysgalactiae subspecies equisimilis*

### Typing of porcine reproductive and respiratory syndrome virus

All 16 cases positive for PRRSV were typed as PRRSV-1 by PCR. Of the two cases in which accompanying material from the sow tested positive for PRRSV, one was typed as PRRSV-1 and one tested positive for both PRRSV-1 and PRRSV-2.

**Fig. 1:**
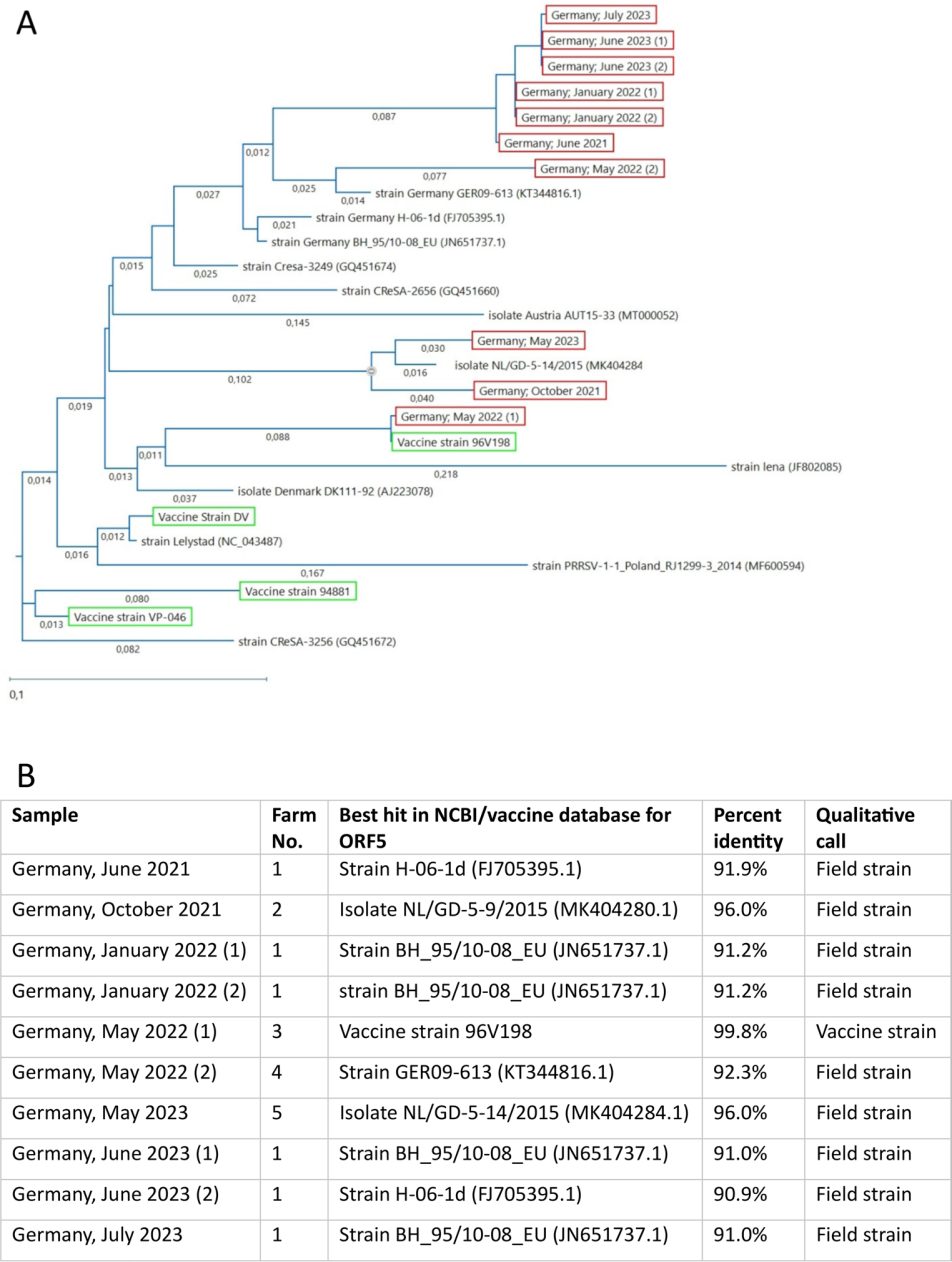
**A**: Phylogenetic tree of PRRSV sequences obtained from Sanger sequencing of field samples and selected reference sequences. The tree was constructed using the Randomized Accelerated Maximum-Likelihood (RAxML) method with 100 bootstrapping iterations. Uncorrected pairwise distances are displayed on the branches. The samples are highlighted with a red box. The vaccine strains are highlighted with a green box. For all published sequences, NCBI accession numbers are noted in brackets after the strain name. **B**: Farm numbers, best hits in local vaccine database or NCBI nt database with respective percent identities and qualitative calls (Vaccine or Field strain) for the strains sequenced and displayed in A. Percent identities were determined by nucleotide BLAST (Altschul et al. 1990)

In the ten cases positive for PRRSV-1 with a Ct value below 30, open reading frame (ORF) 5 was sequenced. In one case the sequenced sample showed a high percentage of identity with a vaccine strain (99.8% identity to strain 96V198). In all other cases, samples displayed percentage identities below 90% to ORF 5 sequences of vaccine strains and percentage identities between 90% and 96% to sequences deposited in the NCBI nucleotide database. Notably, six of these cases originating from the same farm within a period of two years showed high percentage identities ranging from 98.9 to 100% among each other, forming a cluster in the phylogenetic tree (Fig. 1).

## Discussion

Routine diagnostic submissions represent a valuable source of field data; however, they also have inherent limitations that must be considered when interpreting results. In most evaluations about 60–80% of abortions are allocated to non-infectious factors due to negative diagnostic results. Detection rates in our study are comparable with others (Nathues et al. 2011; Donneschi et al. 2024). In 19.1% of the abortion cases, at least one pathogen from the key panel was detected.

In an Italian study detection rates were higher (59%) due to a combination of direct and indirect methods and inclusion of a different panel of pathogens (Salogni et al. 2016). In contrast to the cited study, which is based on a standardized sampling protocol, our study presents diagnostic investigations performed within the framework of routine laboratory diagnostics and were therefore influenced by the specific requests of the submitting veterinarians. Consequently, not all abortion materials were examined for the same panel of pathogens and samples from the sow for indirect methods were rarely (*n* = 11) available. The diagnostic approach reflects real-world practice rather than a standardized research protocol. This heterogeneity may have resulted in an under- or overrepresentation of certain pathogens and limits direct comparison of detection frequencies between agents.

In the diagnostic approach in our study tissues were pooled with respect to the expected highest load for the respective pathogen. Pooling of just one organ might have improved sensitivity (Kreutzmann et al. 2024). However, separate testing of the organs results in higher cost for the practitioner.

In this study, PRRSV-1 field strains were identified by sequencing of ORF5 in nine out of ten cases, indicating potential clinical relevance (Fig. 1). Unfortunately, metadata about vaccination schemes and internal biosecurity on respective farms are lacking. Recent reports suggest comprehensive sequencing of the entire PRRSV genome to detect recombinant strains (Kvisgaard et al. 2020; Vandenbussche et al. 2021).

For PCV2-associated reproductive disease (PCV2-RD) the diagnostic triad (reproductive disorders, myocarditis, at least moderate viral loads in heart tissue) proved to be unsuitable and a threshold of 10^9^ PCV2 genome equivalents (GE)/g heart tissue irrespective of histological findings was recommended for diagnostic of PCV2-RD (Unterweger et al. 2021; Reif et al. 2022). Systematic prevalence studies revealed that more than 20% of aborted fetuses are positive for PCV2 but with low pathogen loads, so that this agent cannot be considered as the etiological pathogen in most cases (Reichl et al. 2026). Among the six PCV2-positive cases in our study, four showed very low CT values (< 10), reflecting high viral loads and suggesting clinical relevance.

The pathogenic potential of PCV3 and *M. suis* is not completely understood, because both are also found in pigs lacking specific clinical signs (Bordin et al. 2021) and in some farms with high prevalences (Ngo et al. 2024). High detection rates for PCV3 were detected in mummified fetuses, but in 93% of the cases also other pathogens were found (Dal Santo et al. 2020).

All isolated bacteria in this study can potentially cause abortions but are also commonly found in swine farm environments, so that the risk of contamination must be minimized by proper sampling.

In this study, no relevant pathogens were detected in 75.4% of cases. It cannot be excluded that further pathogens were present in the samples, for which no testing was commissioned by the customer or for which no specific PCR assay is available. In addition, several pathogens might interact and lead to reproductive problems in a synergistic way. The statistical issue of multiple testing must be considered for the presented data. Although no repeated testing of the same target pathogen was performed in the samples, multiple testing for different pathogens increased the chance for false positives purely by chance. The simultaneous testing for multiple pathogens per case introduced a multiple comparisons problem, increasing the family-wise error rate and thus the probability of obtaining false-positive results. Therefore, diagnostic results should be interpreted cautiously and in the context of clinical history and the known pathogenic significance of the detected agents.

Over the last decade, the untargeted diagnostic approach of metagenomic next-generation sequencing (NGS) has gained increasing importance (Van Borm et al. 2014; Gagnon et al. 2021). In contrast to PCR methods, NGS is not based on pathogen-specific primers and probes and has the potential to discover unknown pathogens. The detection of PCV-3 and other ssDNA viruses linked to reproductive failure through metagenomic analyses highlights the potential of NGS in diagnostic advancements (Palinski et al. 2016; Liu et al. 2026).

New diagnostic approaches could be based on metagenomic NGS in case the costs for these investigations are reduced and studies regarding the applicability of pooling of different tissues will be performed (Van Borm et al. 2014; Gagnon et al. 2021).

Until standardized and cost-effective NGS protocols are available, the first step for diagnostic in abortions should remain diagnostic of the key panel pathogens mentioned here. Although higher detection rates for PCV-2 and PRRSV have been demonstrated in separate pools of thymus and heart than in pluck pools of both organs (Kreutzmann et al. 2024), pooling of different organs from up to five fetuses is common practice for cost-efficient diagnostics and should be based on the tissue tropism of the respective infectious agents. All aborted fetuses should be included in the examination due to the unequal distribution of pathogens within the aborted material (Harding et al. 2017). Bacterial examination can be restricted to stomach content and fetal organs, because this material is less exposed to contamination by the environment. Bacteriological diagnostics should ideally be accompanied by a histological examination of lung and placenta allowing the differentiation of infection with clinical impact from contamination.

## Conclusions

Based on the findings presented here, approximately 20% of all abortions are caused by one of the key pathogens (PRRSV, PPV, PCV2, *Leptospira* and Chlamydiaceae spp.) or mixed infections.

The substantial number of negative cases suggests that abortions were attributed primarily to non-infectious factors. However, the detection of pathogens outside the key panel in 7% of the cases underscores the potential benefits of a more comprehensive diagnostic approach, such as metagenomic next-generation sequencing.

## Supplementary Information

Below is the link to the electronic supplementary material.

Supplementary Material 1

## Data Availability

The datasets generated and/or analyzed during the study are not publicly available owing to the proprietorship of SAN Group Biotech Germany GmbH but are available upon reasonable request.
