# Supplementary material for: Retrospective analysis of infectious agents in swine abortion materials in the years 2021 to 2023
Source: Vet Res Commun. 2026 May 29;50(4):357. doi: 10.1007/s11259-026-11252-x (PMC13221374; doi:10.1007/s11259-026-11252-x)
Supplement: Supplementary file 1 — Supplementary Material 1 [file 11259_2026_11252_MOESM1_ESM.docx]

Supplementary table 1: Sample compositions based on the pathogen of interest. Organs were pooled from a maximum of 5 animals.

| **Targeted Pathogen** | **Pooled tissues/organs** |
| --- | --- |
| *African swine fever virus* | Lung, Thoracic fluid, Spleen, Thymus |
| Chlamydiaceae spp. | Lung, Liver, Placenta |
| *Erysipelotrix rhusiopathiae* | Lung, Liver, Placenta |
| *Influenzavirus A* | Lung, Thoracic fluid, Spleen, Thymus |
| *Leptospira* | Lung, Liver, Kidney, Placenta |
| porcine circovirus 2 | Heart, Thoracic fluid |
| porcine circovirus 3 | Heart, Thoracic fluid (in few cases additionally lung and spleen) |
| porcine parvovirus | Lung, Placenta, Thymus |
| Porcine Reproductive and Respiratory Syndrome Virus | Lung, Thoracic fluid, Spleen, Thymus |

Supplementary table 2: Commercial PCR kits used for detecting pathogens of interest.

| **Targeted Pathogen** | **Used PCR kit** | **Manufacturer** |
| --- | --- | --- |
| *African swine fever virus* | Kylt® ASF | SAN Group Biotech Germany GmbH, Höltinghausen, Germany |
| Chlamydiaceae spp. | Kylt® Chlamydiaceae Screening | SAN Group Biotech Germany GmbH, Höltinghausen, Germany |
| *Erysipelotrix rhusiopathiae* | Kylt® Erysipelotrix rhusiopathiae | SAN Group Biotech Germany GmbH, Höltinghausen, Germany |
| *Influenzavirus A* | Kylt® Influenza A | SAN Group Biotech Germany GmbH, Höltinghausen, Germany |
| pathogenic *Leptospira* | Kylt® Leptospira, pathogenic | SAN Group Biotech Germany GmbH, Höltinghausen, Germany |
| porcine circovirus 2 | Kylt® PCV2 | SAN Group Biotech Germany GmbH, Höltinghausen, Germany |
| porcine circovirus 3 | Kylt® PCV3 | SAN Group Biotech Germany GmbH, Höltinghausen, Germany |
| porcine parvovirus | Kylt® porcine parvovirus (PPV) | SAN Group Biotech Germany GmbH, Höltinghausen, Germany |
| Porcine Reproductive and Respiratory Syndrome Virus | Applied Biosystems™ VetMAX™ PRRSV EU & NA 2.0 | Life Technologies GmbH, Darmstadt, Germany |
